# Personalized Treatment of Head and Neck Cancers: Role of Functional Imaging and AI

**DOI:** 10.3390/cancers18121954

**Published:** 2026-06-16

**Authors:** Joran Tanghe, Rüveyda Dok, Sandra Nuyts

**Affiliations:** 1 Laboratory of Experimental Radiotherapy, Department of Oncology, KU Leuven, 3000 Leuven, Belgium; 2 Department of Radiation Oncology, Leuven Cancer Institute, University Hospitals Leuven, 3000 Leuven, Belgium

**Keywords:** magnetic resonance imaging, positron emission tomography, radiomics, deep learning, head and neck squamous cell carcinoma, chemoradiotherapy, review

## Abstract

Chemoradiotherapy plays an important role in the management of locally advanced head and neck squamous cell carcinoma. Unfortunately, a substantial fraction of patients experience treatment failure, while others suffer from significant treatment-related toxicity caused by intensive chemoradiotherapy regimens. This underscores the need for new biomarkers that can accurately capture the biological tumor heterogeneity and guide personalized therapy. Functional imaging combined with AI-based approaches such as radiomics and deep learning may offer a promising strategy for treatment stratification. However, a substantial number of challenges remain before clinical implementation can be achieved. Therefore, this review proposes a biology-driven framework for AI analysis of functional imaging in head and neck squamous cell carcinoma. In addition, it emphasizes the need for clinically oriented validation strategies to facilitate the translation of stratification models into clinical management.

## Simple Summary

Head and neck cancer is a heterogeneous disease, meaning that tumors differ substantially between patients. As a result, the treatment of head and neck cancer is associated with considerable toxicity and high rates of treatment failure. Therefore, there is a strong need to personalize treatment based on the biological characteristics of the individual tumor. Functional imaging techniques combined with AI approaches may help achieve this goal by identifying imaging patterns associated with the underlying tumor biology and treatment resistance. Consequently, AI methods could support treatment stratification and reduce toxicity and treatment failure. However, implementing these novel tools into clinical practice remains difficult. To address this, this review stresses the importance of constructing AI tools based on tumor biology and evaluating them using clinically relevant measures to facilitate translation into routine clinical management of head and neck cancer.

## 1. Treatment Resistance and Biological Heterogeneity in HNSCC

Head and neck squamous cell carcinoma (HNSCC), with approximately 890,000 new diagnoses and 460,000 deaths annually, is the sixth most common cancer in the world [1]. Approximately 60% of patients present with locally advanced disease, which is treated with surgery and adjuvant chemoradiotherapy (CRT) or CRT alone [2,3]. The CRT regimen often consists of 70 Gy radiotherapy (RT) and concomitant 3-weekly cisplatin with a dose of 100 mg/m^2^ [2]. The current CRT regimen, however, can be associated with substantial toxicity [4]. Additionally, 50% of patients with locally advanced disease experience treatment failure, either through locoregional recurrence (LRR) or distant metastasis (DM) [2]. These shortcomings highlight the need for prognostic factors that can identify patients at risk of treatment failure.

Resistance to CRT can be driven by several biological processes, including hypoxia, the presence of cancer stem cells (CSCs), cell death evasion, metabolism, and enhanced DNA damage repair [5,6,7,8,9]. Hypoxia, or low oxygen levels, is a common feature in solid tumors, including HNSCC, and is one of the most extensively studied biological processes associated with CRT response [10,11]. Hypoxia can be chronic, or diffusion-limited, due to insufficient oxygen supply, and acute, or perfusion-limited, due to abnormal tumor vasculature. These forms often coexist and are heterogeneously distributed within tumors. The relevance of hypoxia is supported by strong radiosensitization of oxygen, resulting in more effective CRT in well-oxygenated cells compared to hypoxic cells, particularly at oxygen levels below 0.5%. This effect is expressed as the oxygen enhancement ratio and is explained by the stabilization of radiation-induced free-radical DNA damage, leading to at least a two-fold increase in lethal DNA lesions under normoxic conditions [11,12,13]. Furthermore, through activation of hypoxia-inducible factors, cells adapt to hypoxic conditions by altering genes involved in survival, angiogenesis, metabolism, and invasion, thereby promoting genetic instability, metastasis, and therapy resistance [14,15].

Current prognostic factors such as the TNM stage, smoking status, and human papillomavirus (HPV) status in oropharyngeal cancer do not fully capture the biological processes driving treatment resistance in HNSCC. Therefore, additional prognostic biomarkers are needed to improve the prediction of treatment response and treatment failure. Functional imaging provides biological information beyond conventional anatomical imaging and may help overcome these limitations by capturing biological processes that drive treatment resistance.

The extraction of biological information from functional imaging may be enhanced by radiomics and deep learning (DL) [16]. Both methods capture information not discernible to the naked eye and may further unravel tumor heterogeneity [17,18]. Radiomics achieves this by the extraction of a vast amount of quantifiable features, whereas in DL, the features are automatically derived from the images.

However, translation of AI tools into clinical practice remains challenging. Generalizability issues, limited interpretability of radiomics and DL, and a lack of external validation constitute substantial hurdles towards clinical translation [16,17].

Therefore, this review covers how functional imaging combined with radiomics and DL can improve prognostication, interpretability, and clinical translation of prediction models in HNSCC. In this review, we propose a biology-driven framework where ROI definition, timing of evaluation, and imaging modality are guided by the biological mechanisms underlying treatment resistance. In addition, we will discuss current validation practices and their limitations and emphasize the need for calibration and clinical utility metrics to enable clinically meaningful evaluation of prediction models.

## 2. Functional Imaging, a Window into the Tumor Biology

### 2.1. MRI

To assess the primary tumor (PT) as well as metastatic lymph nodes (LN) in HNSCC, either contrast-enhanced computed tomography (CT) or magnetic resonance imaging (MRI) is recommended [2]. MRI offers superior soft tissue contrast and is better suited to identify some therapeutically relevant factors such as perineural spread and invasion of the bone marrow [19,20]. An additional advantage of MRI is the potential to include functional imaging modalities such as dynamic contrast-enhanced (DCE) MRI and diffusion-weighted (DWI) MRI [20].

DCE-MRI is an optional imaging modality for the management of HNSCC [20]. It provides information on tumor perfusion, vascular permeability, and the extravascular, extracellular space. The parameters K^trans^, V_e_, and blood volume (BV) capturing this information are determined using the pharmacokinetic extended Tofts model. As shown in Table 1, K^trans^ is the volume transfer rate of the contrast agent between the blood plasma and the extracellular, extravascular space. V_e_, on the other hand, quantifies the size of the extracellular, extravascular space. The extended Tofts model also accounts for the blood volume (BV), which may be substantial in tumors [21].

Adequate blood supply to the tumor is imperative for tumor growth. Therefore, highly vascularized tumors, due to the potential for rapid proliferation, are often considered to be aggressive [25]. Additionally, these fast-growing tumors, due to chaotic angiogenesis, often have less mature blood vessels with increased permeability [25]. The increased tumor perfusion and permeability associated with rapidly proliferating tumors can be captured by K^trans^. Furthermore, rapid proliferation is also associated with increased cellularity and low V_e_. Therefore, the combination of K^trans^ and V_e_ can be used to estimate the proliferative ability of tumors. In line with this, Yang et al. (2024) reported that K^trans^ and V_e_ could be potential biomarkers for Ki-67, a known marker for proliferative ability [22].

Low K^trans^ and BV could be indirect measures of tumor hypoxia. In line with this, Cao et al. (2008) and Wei et al. (2024) found that a low BV, potentially reflecting hypoxia, is a predictor for local treatment failure [23,24].

DCE-MRI may be used to approximate proliferative ability as well as hypoxia. However, careful interpretation of the context-dependent DCE-MRI parameters K^trans^ and BV is needed. Moreover, the clinical translation of DCE-MRI is limited by factors such as lengthy image acquisition [20,21].

Although DWI-MRI is not a standard imaging modality for the clinical management of HNSCC, DWI-MRI is increasingly used for LN diagnosis as well as recurrence detection [2,19,20]. DWI-MRI reflects the diffusion of water molecules, which is related to cellularity, and is commonly evaluated using the apparent diffusion coefficient (ADC) [26].

The ADC is influenced by cellular organization, and therefore, DWI-MRI may help to uncover the microstructure of tumors [27]. The relationship between ADC and cellular organization was used by de Perrot et al. (2017) to identify microstructure differences between HPV positive and HPV negative tumors [28]. The authors reported that HPV-positive tumors had significantly lower mean and median ADC values, while ADC kurtosis and skewness were significantly increased. Additionally, they hypothesized that these imaging findings may reflect the histological organization of HPV positive tumors into tightly packed, homogeneous cell clusters. These findings were further strengthened by Suh et al. (2020), who also demonstrated that ADC maps can be used for HPV prediction [29].

Due to the importance of hypoxia in treatment failure, the potential of ADC as a hypoxia marker has also been evaluated. Han et al. (2025) reported that hypoxic nodes, as detected by FMISO-PET, have a significantly higher pretreatment mean ADC [30]. This may be explained by the presence of necrosis. However, this association was not observed mid-treatment. In contrast, ADC skewness was significantly higher in hypoxic tumors before and during treatment. This indicates that the distribution of the ADC values may reflect tumor heterogeneity and could be important for hypoxia and treatment failure prediction. Similarly, Scalco et al. (2016) demonstrated that pretreatment nodal ADC was significantly higher for patients with treatment failure [27]. They hypothesized that high pretreatment ADC values are related to hypoxia-induced necrosis, which aligns with the findings of Han et al. (2025) [30]. However, they did not identify any significant relationships between mid-treatment ADC values and treatment failure.

DWI-MRI does not require contrast injection, is faster to acquire than DCE-MRI, and reflects cellular organization, which may facilitate the clinical translation of DWI-MRI biomarkers [20,28]. However, using DWI-MRI as a hypoxia marker requires caution, as it provides an indirect assessment of hypoxia through necrosis.

### 2.2. PET

The anatomical imaging modalities CT and MRI are preferred for assessment of the PT. However, for the staging of the LN as well as the detection of DM, ^18^F-fluorodeoxyglucose (FDG) positron emission tomography (PET)/CT is the modality of choice [2,19]. Moreover, the use of FDG-PET/CT is recommended for treatment response assessment at 10–12 weeks after CRT completion [20]. In clinical practice, FDG-PET is almost always combined with CT, which provides anatomical guidance [19]. In addition to FDG, FLT and FMISO are interesting experimental PET tracers for the assessment of biological factors relevant for treatment failure [31,32].

At the moment, FDG is the only PET radiopharmaceutical routinely used in the management of HNSCC [2]. FDG, similarly to glucose, is taken up by cells through glucose transporters. In the cell, FDG is phosphorylated, after which it cannot be further metabolized through glycolysis and remains trapped [33]. FDG-PET can differentiate between tumor and normal tissue through the metabolic shift from oxidative phosphorylation to glycolysis, resulting in increased tumoral glucose uptake and FDG-PET signal [10]. However, various biological processes, such as the cellular proliferation rate and the presence of hypoxia, influence tumor glycolysis and the resulting FDG uptake [33].

FDG-PET, just as DCE- and DWI-MRI, has been investigated as a potential hypoxia marker. Under hypoxic conditions, glycolysis is generally stimulated, which is reflected by an increased FDG-PET signal [10]. Therefore, Crispin-Ortuzar et al. (2017) reported that FDG-PET combined with contrast-enhanced CT reflects hypoxia, as identified by FMISO-PET [34]. They hypothesized that high FDG-PET voxel intensities may represent hypoxic areas with increased glycolysis. Additionally, according to the authors, contrast-enhanced CT is beneficial for hypoxia prediction, as it may capture tumor perfusion. In line with these findings, Lafata et al. (2021) showed that high and heterogeneous FDG uptake is related to treatment failure [35]. They hypothesized that areas with a high FDG-PET signal could reflect hypoxia. Similarly, Martens et al. (2020) reported that heterogeneity within high FDG-PET voxel intensities was associated with treatment failure [36]. However, the authors of this study chose not to hypothesize which biological processes drive the high FDG uptake.

Various studies have reported the potential of FDG-PET for treatment failure prediction. However, more research is necessary to elucidate the relationship between FDG-PET imaging features and the underlying tumor biology beyond hypoxia. This is particularly important given the multifaceted nature of FDG-PET, which reflects a range of biological processes.

All previously discussed functional imaging modalities have been applied to predict the presence of hypoxia. This emphasizes the need for an imaging modality that can provide a more direct assessment of tumor hypoxia. FMISO is the most extensively studied PET radiopharmaceutical for evaluating hypoxia. For various tumor types, including HNSCC, it has been shown that accumulation of ^18^F-fluoromisonidazole (FMISO) PET reflects hypoxia [37].

FMISO, because of its lipophilic nature, can easily cross tissues and the cell membrane through passive diffusion [37]. Therefore, FMISO is less dependent on the blood supply than other PET tracers such as FDG, which is essential to accurately capture hypoxia [38]. After FMISO has entered the cell, FMISO undergoes a reduction, which, under normoxic conditions, is rapidly reversed by reaction with oxygen [37]. However, under hypoxic conditions, at a partial pressure of oxygen of ≤5–10 mmHg, insufficient oxygen is available, and FMISO is further reduced to amino-FMISO, FMISO-macromolecule complexes, and glutathione conjugates of amino-FMISO, which are trapped inside the cell [38,39]. Therefore, FMISO facilitates assessment of cellular hypoxia. However, some non-specific uptake of FMISO in normoxic tissues leads to images with limited contrast between normoxic and hypoxic regions. As a result, the tumor FMISO signal is often evaluated relative to the activity in the blood, the tumor-to-blood ratio (TBR), or normoxic muscle tissue, the tumor-to-muscle-ratio (TMR). It has been reported that a TBR ≥ 1.2–1.4 and a TMR ≥ 1.6 are indicative of hypoxia [40].

Various studies have evaluated the potential of FMISO-PET for hypoxia-based prognostication. Sörensen et al. (2019) demonstrated that the heterogeneity of tumor hypoxia is significantly lower for patients responding to CRT [31]. This lower heterogeneity may be explained by smaller and more homogeneous hypoxic regions. Additionally, Carles et al. (2021) reported that increasing heterogeneity inside the hypoxic region is predictive of treatment response [41]. The improved response with increasing heterogeneity inside the hypoxic tumor region may be driven by reoxygenation.

FMISO-PET is currently the functional imaging modality that can provide the most accurate estimation of hypoxia in HNSCC. Its potential to capture cellular oxygenation and the associated risk of treatment failure has been suggested [31,37,41]. However, more evidence on its performance as a prognostic tool is required. Furthermore, FMISO-PET remains an experimental radiopharmaceutical, so at this moment, it is not readily available for application in the management of HNSCC [31].

^18^F-fluorothymidine (FLT) is another experimental PET radiopharmaceutical of interest, which is currently not used in clinical management of HNSCC [32]. The FLT tracer can be used to assess the proliferation rate of cells [42]. To allow proliferation, an adequate supply of deoxynucleotides, including thymidine, is required. Thymidine can either be synthesized de novo through the endogenous pathway or can be taken up by the cells through the exogenous DNA salvage pathway using nucleoside transporters [43]. FLT, a thymidine analogue, enters the cell through the exogenous pathway and is phosphorylated by thymidine kinase-1 (TK1) [44]. Phosphorylated FLT is trapped inside the cell but is not incorporated into the DNA. The uptake and trapping of FLT are thus regulated by TK1, an enzyme that is upregulated during the S-phase of the cell cycle [32].

Ulrich et al. (2019) reported that the FLT proliferation assessments can be used to predict treatment failure [42]. They found that tumors with a large and heterogeneous proliferative zone have a significantly worse prognosis. However, in some cases, FLT-PET may not reliably reflect the proliferative ability of the imaged tissues. The first potential cause is that the fraction of thymidine entering the cell through the exogenous and endogenous pathway is dependent on the individual tumor, and thus FLT may fail to provide an accurate estimate of cellular proliferation [43]. Secondly, FLT is metabolized in the liver to FLT-glucuronide, which is not taken up by the cells. After one hour, up to 30% of the FLT signal arises from the FLT-glucuronide, which is not related to cellular proliferation [44].

FLT-PET has been reported to be associated with proliferative ability and treatment failure in HNSCC. Additionally, it provides a more direct assessment of the proliferative ability than DCE-MRI. However, under certain conditions, FLT fails to effectively capture the proliferative ability [32]. Therefore, more research is required to establish FLT-PET as a reliable biomarker for cellular proliferation.

## 3. AI Analysis of Functional Images

### 3.1. Methodology

Radiomic analysis, first introduced by Lambin et al. (2012), is based on the extraction of a large number of quantitative features from medical images [45]. The features capturing image intensity, shape, and texture are not discernible through visual analysis and potentially reflect the underlying biology of heterogeneous tumors [18,45]. Radiomics has significant potential for the personalized management of HNSCC, as imaging is an important part of the CRT workflow.

The radiomics pipeline for prognostication consists of 5 main steps: segmentation of a region of interest (ROI), image preprocessing and harmonization, feature extraction, feature selection, and outcome prediction [16]. First, an ROI is segmented to avoid the inclusion of noise from irrelevant structures. Currently, ROIs are most often delineated manually. However, this process is time-consuming and subject to variability. Therefore, automated DL-based ROI segmentation is gaining interest. Additionally, preprocessing and harmonization are essential to account for various sources of technical variation, such as imaging protocols and scanners used. This is paramount to avoid confounding of the true biological feature differences [46]. The third step of the pipeline is the actual extraction of radiomic features. Feature extraction should be performed using algorithms compliant with the Image Biomarker Standardization Initiative (IBSI) [47,48]. This will ensure reproducible feature definitions. Commonly, a large number of features is extracted, necessitating careful feature selection. The selected features need to be stable (e.g., independent of minor segmentation differences), non-redundant (the features should carry independent information), and relevant for the outcome [16,18]. Finally, the selected features are used for outcome prediction using machine learning models.

The functional imaging modalities discussed before enable assessment of the spatial heterogeneity of resistance-related biological processes. Therefore, functional imaging is highly synergistic with radiomics, which captures the tumor heterogeneity [45]. Even though a minority of studies (e.g., Mori et al. (2023) and Sheng et al. (2025)) reported limited added value of functional radiomic imaging features (mainly FDG-PET features) compared to anatomical imaging features [49,50], the majority of investigations demonstrated that functional radiomic features are important for prognostication in HNSCC [51,52,53]. This does not mean that anatomical images are not important for prognostication, as there is substantial evidence indicating that models combining anatomical and functional imaging features outperform single modality models [54,55]. This emphasizes the complementary potential of functional and anatomical imaging for radiomic analysis.

In recent years, DL radiomics has gained increasing attention, partly due to the expectation that DL may outperform handcrafted radiomics, as these may fail to capture all intricacies of the tumor biology [56]. DL radiomics differs from handcrafted radiomics in that relevant features are automatically learned from images, instead of being predefined [16]. As a result, DL has the potential to capture features that cannot be described by handcrafted radiomic feature definitions, potentially revealing more information about the underlying tumor biology [57,58]. However, DL typically requires more data, and in a medical setting, generating enough high-quality data can be challenging. This may be alleviated in part through transfer learning, in which a DL model pretrained in a related setting is used for a different task [56].

Currently, DL radiomics is dominated by convolutional neural networks (CNNs) and vision transformers (ViTs) [59]. Examples of common CNNs that have been used for prognostication in HNSCC include ResNet and DenseNet [52,60]. By combining convolutional and pooling operations, CNNs are highly proficient in extracting hierarchical features from images [61]. In consecutive layers, more complex features are learned, and deeper features may be related to the underlying tumor biology [62]. However, CNNs are less efficient in capturing contextual information. ViTs, which are more appropriate to include long-distance information, have, in some cases, outperformed CNNs [59]. ViTs analyze images in patches, and self-attention mechanisms are used to model relationships between different parts of the images. Although ViTs are more suited to capture contextual information, CNNs remain more effective for local feature extraction and are more data-efficient [59]. Therefore, novel architectures, such as TransRP used by Ma et al. (2024), combine CNNs with ViTs for context-aware local feature extraction [63].

The DL radiomics pipeline is similar to the handcrafted radiomics pipeline. In DL, a segmented ROI may be used, but in recent studies, DL radiomic features derived from entire images have also been investigated. Furthermore, image preprocessing and harmonization are also essential in DL. However, the final steps are combined in an end-to-end framework, as the DL network extracts the most important features and uses them for treatment failure prediction [16,52].

Due to its ability to capture complex relationships, DL radiomics analysis of functional images can potentially facilitate biology-driven prognostication [56]. In HNSCC, most DL-based prognostication has been performed using FDG-PET. The dominance of FDG-PET in the field of DL radiomics can be partly explained by the availability of a multicenter dataset of approximately 850 FDG-PET images made available through the HECKTOR 2022 challenge [64]. Le et al. (2022) showed that FDG-PET imaging was beneficial for LRR but less effective for DM prediction [65]. Furthermore, just as for handcrafted radiomics, de Biase et al. (2023) and Wang et al. (2025) confirmed the complementary nature of FDG-PET and CT and thus of functional and anatomical imaging [60,66]. Even though DL studies using functional imaging modalities other than FDG-PET are scarce, Tomita et al. (2022) reported the value of mid-treatment DWI-MRI for prognostication [67].

### 3.2. Biology-Driven ROI Definition

#### 3.2.1. Primary Tumor and Lymph Node Radiomics

Treatment failure can manifest itself as local recurrence (LR) in the PT, regional recurrence (RR) in the LN, or DM [2]. Many radiomic studies are focused only on the PT. It is clear that the PT is important for LR, but considering the PT alone for RR and DM prediction may be insufficient. Although T-stage has been associated with an elevated risk of DM, other factors such as the N-stage and the presence of extranodal extension are more important [68]. This highlights a potential limitation of radiomics studies relying only on the PT in predicting treatment failure.

Tomita et al. (2022) evaluated the predictive performance of PT radiomics for LR only [67]. Other studies extended PT radiomics to evaluate treatment failure beyond LR. For example, Wei et al. (2024) reported a c-index of 0.77 for LRR and 0.695 for DM using DCE- and DWI-MRI features [24]. Le et al. (2022) demonstrated similar predictive performances of FDG-PET/CT radiomics for LRR and DM [65]. The final model for LRR contained PET and CT features. In contrast, for DM, the addition of PET features resulted in a loss of performance, suggesting that only the morphology (volume) of the PT is relevant for DM. Additionally, several studies (e.g., Jian et al. (2025) and Peng et al. (2019)) have reported that both anatomical and functional PT features may be used to capture treatment failure in general [55,69].

The literature suggests that PT radiomics can be used to predict DM and treatment failure in general. This may be explained by the importance of the tumor grade and T-stage for DM [68]. This hypothesis could then also explain the importance of CT features.

Nevertheless, the majority of patients with locally advanced HNSCC present with nodal metastases [64,70,71]. Moreover, N-stage and presence of extranodal extension are important prognostic factors for RR and DM [68]. Therefore, radiomic analysis combining features from PT and LN may be better suited to capture treatment failure.

Salahuddin et al. (2023) reported that FDG-PET/CT radiomic features extracted from both the PT and the LN resulted in a better estimate of recurrence-free survival than only PT features [71]. In this study, radiomic features were extracted from the PT and LN independently. Using FLT-PET, Ulrich et al. (2019) found that features representing the entire tumoral burden, PT and LN combined in one ROI, are more relevant for treatment failure prediction than only PT features [42]. Although different approaches for LN inclusion were applied, both Salahuddin et al. (2023) and Ulrich et al. (2019) reported the importance of LN for RR and DM prediction [42,71].

The value of functional imaging for assessment of the LN, either on its own or complementary to anatomical imaging modalities, is supported by the literature. Salahuddin et al. (2023) reported that FDG-PET features are more suited to capture the biology of the LN, and CT features are more effective for the PT [71]. The authors explained this finding by hypothesizing that the LN contains more aggressive cancer cells, which may be better characterized by FDG-PET [71]. Similarly, Scalco et al. (2016) showed that the functional imaging (DWI-MRI) features from the LN were most relevant for RR [27]. Furthermore, Vallieres et al. (2017) and Wang et al. (2023), utilizing an ROI consisting of both the PT and LN, found that the combination of functional (FDG-PET) and anatomical images (CT) resulted in the best predictive performance for treatment failure [51,72].

Initial evidence suggests that inclusion of the LN improves RR and DM prediction. However, at this point, there is no consensus on whether the entire tumoral burden should be used as ROI or whether radiomic features should be extracted from the PT and LN independently. A combined ROI may capture total disease burden, but it could mask the heterogeneity inside the LN and PT individually. In contrast, radiomic features extracted from separate ROIs for LN and PT may improve interpretability. However, to date, there is no evidence indicating which approach is optimal for prognostication.

#### 3.2.2. Peritumoral Radiomics

Conventionally, radiomic features are extracted from an ROI consisting of only tumor tissue. However, this approach disregards the peritumoral region (PTR), which is located at the edge of the tumor [50]. The PTR can reflect invasion and stromal and microenvironmental interactions and plays an important role in treatment resistance in HNSCC [10]. Therefore, including the PTR in the radiomic analysis could improve prognostication. However, irrelevant surrounding tissue may increase noise, so the inclusion of relevant PTR and noise needs to be balanced [57].

The potential of PTR in HNSCC has been evaluated mainly using FDG-PET and DL. Ma et al. (2024) used both the full FDG-PET/CT images as well as the manually delineated ROI of the PT and LN as input for a DL radiomics model [63]. Gu et al. (2023) expanded on this investigation by using automatically generated ROIs instead of manual segmentations [52]. Both studies reported that the inclusion of information outside the PT and LN improved performance.

Ma et al. (2024) and Gu et al. (2023) applied binary segmentation masks to focus the models [52,63]. Han et al. (2022) and de Biase et al. (2023) evaluated whether the use of weighted maps, reflecting the probability of the presence of tumor cells, could enhance the information derived from the PTR [58,60]. De Biase et al. (2023) created tumor probability maps (TPM) derived from an automated DL ROI segmentation task [60]. The TPM-based FDG-PET/CT treatment failure prediction showed better or equal performance to a DL network using a manually segmented ROI to guide the model. Similarly, Han et al. (2022), using the RT dose distribution to focus the network, reported that their approach resulted in an improved treatment failure prediction compared to a manually segmented ROI-focused model [58]. These results indicate that weighted maps may aid DL networks to derive the most relevant information from the PTR.

The inclusion of the PTR has also been evaluated in handcrafted radiomics. Sheng et al. (2025) investigated fuzzy radiomics, in which the ROI is weighted with decreasing values from the center towards and beyond the edges of the PT [50]. They reported that this approach improved treatment failure prediction compared to the conventional ROI. However, the weighting approach applied in this study implies that the most important prognostic information is present in the center of the tumor, which is inconsistent with the spatial heterogeneity of tumors [10].

The inclusion of the PTR appears to be beneficial for treatment failure prediction in HNSCC. In addition to the value for prognostication, the applied methodologies could be beneficial to reduce the variability caused by manual segmentations.

#### 3.2.3. Subregion Radiomics

Handcrafted radiomic features capture the extent of tumor heterogeneity but not its spatial organization. This limits their ability to distinguish between diffuse and region-specific variability [73,74]. This highlights a potential drawback of handcrafted radiomics, as subregions with different characteristics are often present in tumors [10,75]. This spatial heterogeneity may be inaccurately captured through radiomic features extracted from the tumor in its entirety. Therefore, to enable analysis of tumor subregions, extraction of radiomic features from subregions may be valuable [75]. It is important to note that subregion radiomics is mainly relevant for handcrafted radiomics, as DL radiomics may implicitly extract features from subregions.

Subregion radiomics using functional imaging has only been investigated with FDG-PET. Nevertheless, Lv et al. (2023) reported that subregion radiomics from PT and LN were more effective to predict treatment failure than features representing the PT and LN in their entirety [76]. This was confirmed in studies by Pan et al. (2023) and Xu et al. (2023), which also found improved performance for the subregion radiomics [73,74]. Xu et al. (2023) reported that a subregion at the border was the most important for treatment failure [74]. They hypothesized that this may indicate aggressive outward tumoral expansion. Another study by Lv et al. (2022) similarly showed the importance of the PT edges for prognostication [77].

Importantly, the biological relevance of subregion radiomics depends on the applied imaging modality. Therefore, FDG-PET, whose signal is an aggregation of biological processes, may not be optimal for subregion radiomics. In contrast, DWI-MRI, FMISO-PET, and FLT-PET, which respectively reflect morphological transformation, hypoxia, and cellular proliferation, may be more suitable for subregion radiomics [27,32,37].

However, these imaging modalities do not capture all mechanisms driving treatment resistance. Other relevant processes such as altered DNA repair capacity or the presence of CSCs should also be considered [10]. Although imaging modalities capturing these processes are currently not used for the management of HNSCC, emerging PET tracers such as ^68^Ga-pentixafor and ^18^F-PARPi targeting CSCs and DNA damage repair processes may offer new opportunities for biology-driven radiomics [78,79].

The available evidence suggests that spatial radiomic analysis may improve prognostication and support biologically informed RT boost strategies [74]. However, evidence remains limited and largely confined to FDG-PET. Future studies should explore subregion radiomics using (a combination of) imaging modalities that more specifically represent treatment resistance.

### 3.3. Treatment Monitoring

Functional imaging and radiomic analysis can be used to assess how the tumor changes during treatment. CRT affects the tumor biology in various ways; hypoxic regions can be reoxygenated, sensitive cells are killed, and radioresistant clones may be selected and drive repopulation [10]. These biological changes are reflected in the tumor heterogeneity and can facilitate treatment monitoring and adaptation [75].

There are two main approaches to evaluating treatment monitoring. Most commonly, the pre- and mid-treatment features are evaluated independently. Using FDG-PET/CT, Lafata et al. (2021) and Starke et al. (2023) compared the value of radiomic features extracted before and after two and three weeks of CRT [35,80]. They reported that the mid-treatment features were more predictive of treatment failure than the pretreatment features. The value of treatment monitoring was also investigated using DWI-MRI. Tomita et al. (2022) identified that radiomic features extracted after four weeks were the only features able to predict treatment failure [67]. In contrast, Scalco et al. (2016) reported that prediction of RR was worse for mid-treatment than for pretreatment radiomic features [27]. A potential explanation for this discrepancy is that Tomita et al. (2022) evaluated the PT, while Scalco et al. (2016) considered only the LN [27,67].

The second strategy is delta-radiomics, in which the evolution of features from pre- to mid-treatment is evaluated. Bollen et al. (2025), using pretreatment and week 4 DWI-MRI images, showed that delta-radiomic features were more effective for LRR prediction than both the pre- and mid-treatment features independently [81]. In contrast, Starke et al. (2023) reported that the delta-radiomic FDG-PET/CT features did not significantly outperform the mid-treatment features [80]. This discrepancy may be explained by several factors. DWI-MRI captures cellular density, which, in part due to rapid apoptosis after CRT initiation, is affected very early on during treatment [82]. By contrast, the FDG-PET/CT signal during treatment and early post-treatment may also be driven by inflammation [33,82]. Therefore, the timing for delta-radiomic analysis should be determined based on the biology reflected by the functional imaging modality.

Mid-treatment radiomic features are promising to capture treatment response. Accurate mid-treatment prediction of treatment failure is important for clinical management of HNSCC, as it may uncover initial treatment resistance and enable adaptive CRT [83]. However, the optimal methodology and timing requires further investigation.

## 4. Barriers to Clinical Translation

The goal of handcrafted and DL radiomic analysis of functional imaging is to develop clinical decision support systems (CDSSs) to facilitate personalized management of HNSCC. Functional imaging-based radiomic analysis has shown promise in response prediction and treatment monitoring in HNSCC in early studies. However, the translation of radiomics research into clinical practice is limited. Development of radiomic CDSS is hindered by the poor interpretability, generalizability, and validation of radiomics research [16].

### 4.1. Interpretability

Decisions made by the model need to be understandable. The interpretability of CDSS is driven by two factors: transparency of the prediction model and feature explainability [84].

Model transparency refers to the extent to which the decision-making process of a model can be understood. Handcrafted radiomics is often regarded as more transparent because the feature selection and machine learning models can be more interpretable. In contrast, in DL radiomics, feature extraction and decision-making are performed end-to-end within a black box. However, black box DL methods often show improved performance [56]. Thus, the transparency and predictive performance may need to be balanced [84].

However, the intrinsic explainability of handcrafted radiomic features is not necessarily better than DL features [84]. In handcrafted radiomics research, regardless of the imaging modality, features often related to global tumor heterogeneity were reported as the most important predictors for treatment failure [31,51]. Even though tumor heterogeneity is a known prognostic factor for treatment resistance, radiomic features capturing heterogeneity cannot inherently be considered explainable, as heterogeneity may be driven by various factors [10,31,41].

The explainability of handcrafted radiomic features can be improved by a more biology-driven ROI definition. Subregion radiomics, particularly when combined with functional imaging modalities, may yield features that are more directly linked to specific biological processes. Separate evaluation of the LN and PT may improve the identification of biological processes driving resistance in the LN and PT individually. In addition, radiogenomics, which studies the correlation between radiomic features and genomic patterns, may help improve the explainability of radiomic features [85].

For DL radiomics, explainability is derived through post hoc interpretability techniques such as GradCAM, which aim to derive the features driving decision-making [63,84]. GradCAM is used to highlight the image regions that contribute most to the treatment failure prediction. Combined with functional imaging, this may provide insight into the biological mechanisms underlying treatment failure. However, post hoc interpretability methods do not always identify the regions that are relevant for decision-making [86]. Therefore, the explainability of DL radiomics is mainly dependent on the accuracy of the post hoc interpretability algorithms. In contrast to handcrafted radiomics, the ROI definition has less influence on the explainability of DL radiomic features, as these are not necessarily calculated for the entire ROI.

However, an important limitation for both radiomics and DL-based imaging approaches is that their features represent aggregate tissue-level measurements. Even when subregion radiomics is applied, imaging features reflect spatially averaged signals rather than direct cell-level or molecular information. Therefore, subregion radiomics may indicate the presence of relevant variation but be unable to determine which cellular populations or molecular mechanisms are responsible for therapy resistance. To address this limitation, subregion radiomics could be integrated with spatially guided molecular profiling approaches [87]. This may help link macroscopic imaging phenotypes to cellular subpopulations and molecular pathways involved in treatment resistance and improve the biological interpretability of AI-derived functional imaging biomarkers.

### 4.2. Generalizability

The generalizability of radiomic findings to independent prospective or multicenter data is essential for clinical translation [18]. Unfortunately, all steps of the radiomic pipeline are associated with challenges that may limit the generalizability of both handcrafted and DL radiomics research [16].

ROI segmentation is, to date, still mainly performed manually, and introduces interobserver variability [16,18]. Various strategies exist to reduce segmentation-based generalizability challenges. The first possibility is the selection of features that are robust under segmentation variations [16]. Second, fuzzy radiomics applying loose ROI boundaries could also reduce interobserver variability [50]. The third strategy is automated segmentation. It has been shown that automated segmentation is accurate and could also be beneficial for prognostication, as it may help focus the DL radiomics network on the most relevant information [16,52]. Finally, DL radiomics can also be performed segmentationless. However, a manually or automatically segmented ROI as guidance is required to avoid the inclusion of noise [60,63].

Radiomic analysis often relies on images acquired with different scanners or imaging protocols. If not accounted for, these factors have a substantial influence on the generalizability of radiomic features. Therefore, careful preprocessing and harmonization are essential. Preprocessing and harmonization may include voxel size harmonization, noise removal, intensity binning, and ComBat harmonization [16]. The applied algorithms can also significantly affect the radiomic features [46]. In this regard, tensor radiomics, as proposed by Salmanpour et al. (2023), in which features are extracted after applying different preprocessing algorithms leading to “flavored” features, presents a promising and innovative technique [88].

For handcrafted radiomics, another source of variability is feature extraction. Mainly in older radiomic studies, features were extracted using in-house-developed algorithms with inconsistent feature definitions [27,89]. This limits the generalizability. However, the introduction of IBSI has resulted in a shift toward more generalizable feature extraction methods [16,47,48].

Even with consistent IBSI-compliant feature definitions, the high dimensionality of radiomics, together with limited medical datasets, remains challenging. To avoid overfitting and false discoveries, selection of stable, relevant, and non-redundant features is essential [16]. However, different feature selection algorithms can identify different sets of features [90]. In addition, small variations in the dataset can lead to the selection of different but correlated features and to changes in the machine learning performance, thereby further limiting the generalizability [90]. To address this issue, factor analysis approaches such as the method proposed by Martens et al. (2020) can be interesting [36]. By reducing groups of correlated radiomic features into a smaller number of factors, this approach may result in the inclusion of broader prognostic information while reducing redundancy. This can further improve prognostication and generalizability.

Finally, regardless of the methodology, dataset quality remains one of the most significant obstacles to the generalizability of radiomic prediction models. Many available datasets are retrospective and small in size, which increases the risk of selection bias. This can result in dataset-specific characteristics instead of true tumor-related features being included in the prediction models [91]. Additionally, a balanced representation of the population is essential to ensure the usability of the CDSS in clinical practice [16,71].

A plethora of factors negatively influence the generalizability of radiomic analysis. Methodological advances combined with the introduction of IBSI and the radiomics quality score (RQS) have enabled the first steps toward standardization and improved generalizability [16,47]. In theory, multiple steps of the radiomic pipeline, such as preprocessing and feature selection, could benefit from standardization. In practice, the optimal methods highly depend on the imaging modality and the tumor type [47]. Consequently, standardization remains difficult, and methodological choices should be clearly justified and based on the characteristics of the dataset.

### 4.3. Validation

Validating the predictive performance and clinical utility of the model is of utmost importance for any CDSS [92]. Internal validation is important, as it gives an initial, optimistic estimate of model performance and enables detection of overfitting [93]. However, it does not capture how a CDSS generalizes to related populations, which is essential for clinical translation. Internal validity is often determined using a holdout set that remained unseen during training, or through cross-validation or bootstrap resampling, which provide a more robust performance assessment [16].

The generalizability of CDSS in related datasets is assessed through external validation. It can be evaluated using data from the same hospital but at a different time (temporal), or using data from different hospitals (geographic) [92]. CDSS in clinical practice will inevitably be applied to temporal or geographic datasets, emphasizing the importance of external validation.

Nevertheless, Table 2 highlights that external validation is lacking in a substantial number of radiomic studies in HNSCC. This can be explained by the difficulty of acquiring temporal and geographic external validation datasets. This underscores the importance of the publicly available HECKTOR 2022 multicenter dataset, enabling external validation and guiding radiomics research toward clinical translation [64].

#### 4.3.1. Discriminative Performance

The most commonly used metrics in internal and external validation of CDSS assess discriminative performance. For classification tasks, the receiver operating characteristic (ROC) curve and associated area under the curve (AUC) are most frequently reported. By contrast, survival models are predominantly evaluated using Harrell’s concordance index (c-index) [92].

The ROC curve is constructed by evaluating the true-positive rate (sensitivity) and false-positive rate (1 − specificity) at different decision thresholds. The AUC summarizes discriminative performance at different levels of sensitivity and specificity, providing a simple measure facilitating comparison between models. However, in clinical practice, model performance across all thresholds is irrelevant, and the AUC does not account for the relative importance of sensitivity and specificity. Depending on the clinical context, one may be far more important than the other, and performance should therefore be evaluated at a clinically relevant operating point [94].

The c-index evaluates the concordance between pairs of patients. In survival analysis, concordance occurs when a lower predicted risk is associated with a later event in a pair of patients. However, due to the rank-based evaluation, a higher c-index is not necessarily associated with improved discriminative performance. For example, tied risk predictions are penalized equally regardless of the magnitude of the associated survival differences, despite implying different discriminative performance. Furthermore, the c-index does not directly capture the sensitivity and specificity of the model, which is essential for clinical evaluation of a model [95].

Table 2 shows that for both internal and external validation, there is an overdependence on discriminative performance metrics. In certain studies, AUC or c-index metrics are compared with those of existing prognostic tools, such as TNM stage or HPV status [69]. However, neither the AUC nor the c-index adequately reflects clinical value. Therefore, such comparisons based only on discriminative metrics cannot be interpreted as evidence of clinical utility. Thus, validation of radiomic CDSS should be expanded beyond discriminative performance metrics.

#### 4.3.2. Calibration

For clinical translation, it is essential that model outputs accurately reflect the true observed probabilities [16]. This is assessed through calibration. For well-calibrated models, the predicted probability corresponds to the observed event rate in groups of patients with a similar predicted risk [92].

The most commonly used calibration metric is the Hosmer–Lemeshow test [96]. However, the test is highly influenced by binning and the dataset size. Additionally, the test does not provide an indication of the magnitude and direction of potential miscalibration. The Cox intercept and slope method may be a more informative approach. This method does not rely on binning, and the intercept and slope quantify the overall miscalibration and direction of the miscalibration, respectively [92,96]. Such information is also relevant for the clinical usage of CDSS.

Even though calibration is valuable during internal and external validation, it is evaluated in less than 50% of CDSS development studies [96]. For internal validation, calibration assessment is particularly relevant for certain machine learning algorithms, such as support vector machines, which are known to be poorly calibrated. During external validation, dataset variability may cause significant miscalibration [92,96]. Substantial miscalibration due to the model or dataset may require recalibration using techniques such as Platt scaling or isotonic regression [96]. Additionally, for both internal and external validation, calibration is essential for assessing clinical utility, as most methods used to evaluate clinical value rely on accurate probability estimates.

#### 4.3.3. Clinical Utility

Translation of a prognostic CDSS into clinical practice requires substantial evidence of its clinical utility. Specifically for personalized management of HNSCC, CDSS should enable stratification into clinically meaningful low- and high-risk groups and provide added value compared to current prognostic tools [16]. In clinical practice, the harm caused by false-positive and false-negative predictions can be substantially different. Therefore, clinical utility metrics need to incorporate the relative importance of the sensitivity and specificity. This is lacking in discriminative performance metrics. Decision curve analysis (DCA) is better suited to assess the clinical decision-making potential of CDSS, as it evaluates the net benefit across potential clinical decision thresholds, weighting sensitivity and specificity [92].

The net benefit, derived using Equation (1), weights the potential benefits and costs resulting from the use of CDSS. The benefit of a model is determined by the true positives. The cost, on the other hand, is determined by weighting the false positives according to the decision threshold, reflecting the balance between the harm caused by false positive and false negative predictions [97]. This makes the method particularly relevant for treatment adaptation strategies. For example, in treatment escalation based on prognostication, a threshold probability of 10% indicates that the benefit of correctly escalating treatment for one patient is considered equivalent to the harm of overtreating nine patients [92].

(1) $$\begin{array}{cc} Net\;benefit & =\frac{True\;positives}{n}-\frac{False\;positives}{n}*\left(\frac{p_{t}}{1-p_{t}}\right),\\ n & =\mathrm{dataset}\;\mathrm{size}\;\mathrm{and}\;p_{t}=\mathrm{threshold}\;\mathrm{probability} \end{array}$$

Depending on the relative importance of the sensitivity and specificity, in clinical practice, only a limited range of thresholds is actually relevant. Comparison of the net benefit of CDSS and that of the current prognostic tools across this range allows assessment of the clinical potential of the CDSS [97]. Furthermore, DCA can be relevant for both internal and external validation. Nevertheless, as shown in Table 2, in the vast majority of radiomics research, clinical utility is not evaluated. Finally, it is important to recognize that DCA is only relevant for calibrated models, as inaccurate risk estimates could lead to misleading conclusions.

## 5. Future Directions

The poor outcomes and treatment toxicity of the current CRT regimen, combined with the heterogeneity of HNSCC, underscore the need for treatment personalization. Radiomics and AI-based analysis of functional imaging can help uncover the mechanisms of treatment resistance and guide patient stratification. Despite this potential, several methodological challenges limit the clinical translation. Further improvements are necessary before these approaches can be implemented in routine clinical practice.

The increased adoption of IBSI and RQS has contributed to the improved generalizability of radiomics research [16,47]. However, as mentioned above, generalizability could significantly benefit from the standardization of preprocessing and the use of standardized imaging protocols within the radiomic pipeline. Therefore, further research should be performed to identify the optimal radiomic pipeline specific to HNSCC. Moreover, the field could benefit from more large multicenter datasets for other imaging modalities beyond FDG-PET. Such datasets can reduce the risk of selection bias, help mitigate overfitting, and facilitate assessment of generalizability through external validation. In addition, CDSSs are characterized by interpretability issues and are predominantly evaluated using discriminative performance metrics alone, which limits their potential as stratification tools for the clinical management of HNSCC [16,18]. Therefore, future radiomics research should focus on bridging this gap by improving interpretability and validation.

Functional imaging in radiomics research has primarily been used to improve prognostication. However, given its relationship with biological heterogeneity, the combination of radiomics and functional imaging also has the potential to improve interpretability. As shown in Table 2, 14 out of 33 studies defined the ROI as the entire PT. Given the importance of the LN and intratumoral heterogeneity in HNSCC, this approach may be insufficient [10,70]. Therefore, future research should consider biology-driven ROIs, including the PTR, subregions related to tumor biology such as hypoxia and CSC, or the LN, to improve the interpretability and prognostic performance of radiomic CDSS.

Additionally, 20 of the 33 studies discussed used only FDG-PET, which is influenced by several biological processes, resulting in a more challenging interpretation of radiomic features. Other imaging modalities, such as FMISO-PET and DWI-MRI, may capture more specific biological processes and should be considered for future radiomics research.

Due to the availability of the HECKTOR 2022 dataset, external validation was performed in almost 15 out of 33 discussed studies [64]. However, the potential of this dataset has not been fully leveraged, as validation in most studies remained limited to discriminative metrics, often focusing on incremental c-index improvements. As shown in Table 2, calibration was only performed in 9 out of 33 studies. Only two studies used calibration slope and intercept, which are most relevant for clinical translation. In addition, clinical utility was evaluated in only three studies, emphasizing the need for improved validation of CDSS for HNSCC. Future radiomics research should move beyond discrimination alone and assess calibration and clinical utility. Moreover, stricter adherence to RQS validation recommendations and established validation frameworks such as the one developed by Steyerberg et al. (2014) may improve the quality and clinical relevance of radiomics-based prediction models [16,92].

## 6. Conclusions

Functional imaging provides biologically relevant information on treatment resistance beyond the conventional prognostic factors. When combined with a biology-driven ROI definition, functional imaging may also improve the interpretability of radiomics. However, radiomics research remains in the early stages of CDSS development, and substantial improvements in generalizability, interpretability, and clinically oriented validation are required before clinical translation.

## Figures and Tables

**Table 1:** Biological meaning and interpretation of DCE-MRI parameters.

DCE-MRI Parameters	Biological Meaning	Interpretation	References
↑ K^trans^	Increased contrast transfer between blood and the extravascular extracellular space.	May indicate increased perfusion and/or vascular permeability, often associated with angiogenesis and immature leaky vessels.	[21,22]
↓ K^trans^	Reduced contrast delivery.	May indicate low perfusion, reduced permeability, or poor vascular function.	[23,24]
↑ V_e_	Increased extravascular extracellular space.	May indicate edema, necrosis, or lower cellular density.	[21,22]
↓ V_e_	Reduced extravascular extracellular space.	Often associated with increased cellularity.	[21,22]
↑ BV	Increased blood volume.	May be associated with improved oxygenation and treatment delivery.	[23,24]
↓ BV	Reduced vascular volume and limited blood supply.	May reflect hypoxic tumor regions.	[23,24]
↑ K^trans^ + ↓ V_e_	Increased perfusion or permeability together with increased cellularity.	Has been associated with higher Ki-67 or proliferative activity in some studies.	[22]
↓ K^trans^ + ↓ BV	Poor perfusion together with reduced vascular volume.	Has been associated with hypoxia-related aggressiveness and potential radioresistance.	[23,24]

**Table 2:** Overview of the studies covering functional imaging radiomics-based prognostication in HNSCC discussed in this review.

Study Details	N	Imaging	Radiomics Methodology	ROI	Target Outcome	Validation			
						Dataset	Discriminative Performance (Value, 95% CI)	Calibration	Clinical Utility
*Primary tumor and lymph nodes radiomics*									
Peng et al. (2019), [69]	707	FDG-PET/CT	DL	PT	Treatment failure	Internal, 10-fold cross-validation (*n* = 237)	CT and FDG-PET: c-index = 0.72 (0.65–0.79); FDG-PET: c-index = 0.68 (0.61–0.76)	Hosmer-Lemeshow test: *p* = 0.44	\
Le et al. (2022), [65]	669 *	FDG-PET/CT	DL	PT	LRR and DM	External, leave-one-institution- out cross-validation (*n* = 669)	DM: CT and clinical, AUC = 0.67 (0.61–0.73) LRR: CT and clinical, AUC = 0.68 (0.65–0.72)	\	\
Xu et al. (2023), [54]	132	FDG-PET/CT; MRI: T1 and T2	Handcrafted	PT	Treatment failure	Internal, hold-out (*n* = 44)	Clinical, FDG-PET, CT and MRI, c-index = 0.76 (0.64–0.87); FDG-PET, c-index = 0.54 (0.40–0.68)	Visual assessment	DCA: for threshold probabilities between 0.1 and 0.5, the best model has a higher net benefit than current biomarkers
Salmanpour et al. (2023), [88]	408 *	FDG-PET/CT	Handcrafted (tensor)	PT	Treatment failure	External, 5-fold cross-validation (*n* = 408)	Tensor radiomics (CT and FDG-PET): c-index = 0.71 Normal radiomics (CT and FDG-PET): c-index = 0.68	\	\
Mori et al. (2023), [49]	66	FDG-PET/CT	Handcrafted	PT	OS	External, hold-out (*n* = 66)	CT and FDG-PET, c-index (95% CI) = 0.75 (0.62–0.85)	\	\
Jian et al. (2025), [55]	126	MRI: T1, T2 and DWI	Handcrafted	PT	Treatment failure	Internal, hold-out (*n* = 38)	T1 and ADC, c-index = 0.79 (0.76–0.81); ADC, c-index = 0.74 (0.71–0.77)	Visual assessment	DCA and clinical impact curve without comparison with current biomarkers
Qiu et al. (2025), [53]	104	MRI: T1, T2 and DWI	Handcrafted	PT	Tumor response	Internal, hold-out (*n* = 31)	T1, ADC and DWI, AUC = 0.89 (0.76–1.0); ADC, AUC = 0.82 (0.67–0.97)	Hosmer-Lemeshow test: *p* = 0.61	DCA: best model showed higher net benefit than current biomarkers
Scalco et al. (2016), [27]	30	CT; MRI: T2 and DWI	Handcrafted	LN	RR	Internal, leave-one-out cross-validation (*n* = 30)	ADC and T2, accuracy = 0.83; ADC, accuracy = 0.80	\	\
Vallières et al. (2017), [51]	300 *	FDG-PET/CT	Handcrafted	PT and LN	LRR and DM	External, hold-out (*n* = 106)	DM: Clinical and CT, c-index = 0.88 LRR: clinical, CT and FDG-PET, c-index = 0.67	\	\
Ulrich et al. (2019), [42]	30	FLT-PET/CT	Handcrafted	PT and LN	Treatment failure	No independent validation set	PT: FLT-PET, c-index = 0.71; PT and LN: FLT-PET, c-index = 0.86	\	\
Martens et al. (2020), [36]	173	FDG-PET/CT	Handcrafted	PT and LN	LRR and DM	External, hold-out (*n* = 71)	DM: FDG-PET, c-index = 0.63 LRR: clinical and FDG-PET, c-index = 0.65	\	\
Salahuddin et al. (2023), [71]	828 *	FDG-PET/CT	Handcrafted	PT and LN	LRR	External, 5-fold cross-validation (*n* = 359)	PT: CT, c-index = 0.63; FDG-PET, c-index = 0.60 LN: CT, c-index = 0.61; FDG-PET, c-index = 0.64 PT and LN: CT, c-index = 0.66; FDG-PET, c-index = 0.62	\	\
Wang et al. (2023), [72]	220 *	FDG-PET/CT	Handcrafted	PT and LN	OS	External, leave-two-institutions-out cross-validation (*n* = 220)	CT, FDG-PET and dose distribution, c-index = 0.82; FDG-PET and dose distribution, c-index = 0.79	Visual assessment	\
Wang et al. (2025), [66]	806 *	FDG-PET/CT	Handcrafted and DL	PT and LN	OS	External, hold-out (*n* = 143)	Handcrafted: CT and FDG-PET, c-index = 0.73; FDG-PET, c-index = 0.67 DL: CT and FDG-PET, c-index = 0.92; FDG-PET, c-index = 0.90	\	\
Han et al. (2025), [30]	94	MRI: T1, T2 and ADC	Handcrafted	LN	RR	Internal, 5-fold cross-validation (*n* = 94)	ADC, AUC = 0.72; T1 and T2, AUC = 0.66	\	\
*Peritumoral radiomics*									
Han et al. (2022), [58]	157 *	FDG-PET/CT	DL	PTR	LRR	Internal, 5-fold cross-validation (*n* = 157)	PTR: clinical, CT and FDG-PET, AUC = 0.89; FDG-PET, AUC = 0.83 No PTR: CT and FDG-PET, AUC = 0.77	\	\
Gu et al. (2023), [52]	886	FDG-PET/CT	DL	PTR	Treatment failure	External, hold-out (*n* = 234)	PTR: clinical, CT and FDG-PET, AUC = 0.73 (0.63–0.83) No PTR: CT and FDG-PET, AUC = 0.70 (0.61–0.80)	Hosmer-Lemeshow test: PTR: *p* = 0.21	\
Ma et al. (2024), [63]	400 *	FDG-PET/CT	DL	PTR	LRR and DM	Internal, hold-out (*n* = 100)	DM: PTR: CT and FDG-PET, c-index = 0.84 (0.75–0.92) no PTR: clinical, CT and FDG-PET, c-index = 0.73 (0.57–0.87) LRR: PTR: clinical, CT and FDG-PET, c-index = 0.67 (0.55–0.80) no PTR: clinical, CT and FDG-PET, c-index = 0.66 (0.52–0.78)	DM: PTR: slope = 1.5, intercept = −0.47 LRR: PTR: slope = 0.78, intercept = 0.14	\
De Biase et al. (2024), [60]	399	FDG-PET/CT	DL	PTR	LR, RR and DM	Internal, bootstrapped (*n* = 100)	LR: CT and FDG-PET, c-index = 0.74 (0.60–0.86); FDG-PET, c-index = 0.70 (0.52–0.86) RR: clinical and FDG-PET, c-index = 0.61 (0.49–0.74); CT and FDG-PET, c-index = 0.56 (0.42–0.71) DM: clinical, CT and FDG-PET, c-index = 0.73 (0.52–0.90); FDG-PET, c-index = 0.62 (0.47–0.78)	LR: slope = 0.84, intercept = 0.12 RR: slope = 0.69, intercept = 0.18 DM: slope = 0.86, intercept = 0.090	\
Sheng et al. (2025), [50]	224 *	FDG-PET/CT	Handcrafted	PTR	Treatment failure	External, hold-out (*n* = 72)	PTR: clinical, CT and FDG-PET, c-index = 0.75 (0.63–0.88) No PTR: clinical, CT and FDG-PET, c-index = 0.71 (0.56–0.88)	\	\
*Subregion radiomics*									
Lv et al. (2022), [77]	806 *	FDG-PET/CT	Handcrafted	Subregions of PT and LN	Treatment failure	External, bootstrapped (*n* = 209)	Subregions: CT and FDG-PET, c-index = 0.64 (0.58–0.72) Entire PT and LN: CT, c-index = 0.58 (0.51–0.66)	\	\
Lv et al. (2023), [76]	642 *	FDG-PET/CT	Handcrafted	Subregions of PT and LN	Treatment failure	External, 20 times 5-fold cross-validation (*n* = 135)	Subregions: CT, c-index = 0.70 (0.69–0.71); FDG-PET, c-index = 0.67 (0.66–0.69) Entire PT and LN: CT and FDG-PET, c-index = 0.67 (0.66–0.68); FDG-PET, c-index = 0.62 (0.61–0.63)	\	\
Pan et al. (2023), [73]	228 *	FDG-PET/CT	Handcrafted	Subregions of PT and LN	LRR	External, bootstrapped (*n* = 98)	Subregions: CT and FDG-PET, c-index = 0.72 Entire PT and LN: CT and FDG-PET, c-index = 0.62	\	\
Xu et al. (2023), [74]	325 *	FDG-PET/CT	Handcrafted	Subregions of PT	Treatment failure	External, hold-out (*n* = 101)	Subregions: clinical, CT and FDG-PET, c-index = 0.72 Entire PT: clinical, CT and FDG-PET, c-index = 0.71	Visual assessment	\
*Treatment monitoring*									
Scalco et al. (2016), [27]	30	Pre- and mid-treatment CT; MRI: T2 and DWI	Handcrafted	LN	RR	Internal, leave-one-out cross-validation (*n* = 30)	Pretreatment: ADC and T2, accuracy = 0.83; ADC, accuracy = 0.80 Delta-radiomics: T2, accuracy = 0.68; ADC, accuracy = 0.64	\	\
Sörensen et al. (2020), [31]	29	Pre- and mid-treatment FMISO-PET/CT; FDG-PET/CT	Handcrafted	PT	OS	No independent validation set	Pretreatment: FDG-PET, no significant split (*p* = 0.86); FMISO-PET, no significant split (*p* = 0.080) mid-treatment (week 2): FMISO-PET, no significant split (*p* = 0.34) Delta-radiomics (week 2–5): FMISO-PET, significant split (*p* = 0.040)	\	\
Carles et al. (2021), [41]	35	Pre- and mid-treatment FMISO-PET/CT	Handcrafted	PT	Treatment failure	External, hold-out (*n* = 10)	mid-treatment (week 2): FMISO-PET, AUC = 0.78 mid-treatment (week 5): FMISO-PET, AUC = 0.79	\	\
Lafata et al. (2021), [35]	64	Pre- and mid-treatment FDG-PET/CT	Handcrafted	PT	Treatment failure	No independent test set	Pretreatment: no significant stratification mid-treatment (week 2): FDG-PET, HR = 7.5 (2.5–22), *p* = 0.020	\	\
Tomita et al. (2022), [67]	70	Pre- and mid-treatment DWI-MRI	DL	PT	LRR	Internal, hold-out (*n* = 21)	Pretreatment: ADC, AUC = 0.63 mid-treatment (week 4): DWI, AUC = 0.77	\	\
Starke et al. (2023), [80]	45	Pre- and mid-treatment FDG-PET/CT	Handcrafted	PT	LRR	Internal, bootstrapped (*n* = 18)	Pretreatment: CT and FDG-PET, c-index = 0.56 (0.24–0.85); FDG-PET, c-index = 0.54 (0.24–0.85) Pre- and mid-treatment (week 0 and 3): CT and FDG-PET, c-index = 0.57 (0.11–0.88); FDG-PET, c-index = 0.70 (0.23–1.0) Delta-radiomics (week 0–3): CT, c-index = 0.80	\	\
Wei et al. (2024), [24]	93	Pre- and mid-treatment FDG-PET/CT; MRI: DCE and DWI	Handcrafted	PT	LRR and DM	Internal, 10 times 5-fold cross-validation (*n* = 93)	LRR: pre- and mid-treatment (week 0 and 2): clinical, FDG-PET, DCE-MRI and ADC, c-index = 0.77 (0.73–0.79) DM: pre- and mid-treatment (week 0 and 2): clinical and DCE-MRI, c-index = 0.70 (0.66–0.73)	\	\
Bollen et al. (2025), [81]	178	Pre- and mid-treatment DWI-MRI	Handcrafted	PT	LRR	Internal, 5-fold cross-validation (*n* = 178)	Pretreatment: clinical and ADC, c-index = 0.56 (0.44–0.66) Delta-radiomics (week 0–4): clinical and ADC, c-index = 0.63 (0.54–0.73)	\	\
Han et al. (2026), [30]	94	Pre- and mid-treatment MRI: T1, T2 and ADC	Handcrafted	LN	RR	Internal, 5-fold cross-validation (*n* = 94)	Pretreatment: ADC, AUC = 0.72; T1 and T2, AUC = 0.66 mid-treatment (week 4): ADC, AUC = 0.72; T1 and T2, AUC = 0.79	\	\

* Data derived from (a subset of) the HECKTOR 2022 dataset [64]. Abbreviations: n = dataset size (training and validation); ROI = region of interest; CI = confidence interval; DL = deep learning; PT = primary tumor; LN = metastatic lymph nodes; PTR = peritumoral region; OS = overall survival; LRR = locoregional recurrence; LR = local recurrence; RR = regional recurrence; DM = distant metastasis; AUC = area under the curve; c-index = Harrell’s concordance index; DCA = decision curve analysis.

## Data Availability

No new data were created or analyzed in this study. Data sharing is not applicable to this article.
